# Sediment sources of Yan'gou watershed in the Loess Hilly region China under a certain rainstorm event

**DOI:** 10.1186/2193-1801-2-S1-S2

**Published:** 2013-12-11

**Authors:** Xue-xuan Xu, Tong-jun Ju, Shi-qing Zheng

**Affiliations:** Institute of Soil and Water Conservation, Chinese Academy of Sciences and Ministry of Water Resources, Yangling, Shaanxi China

**Keywords:** Sediment yield, Land use, Rainfall event, Road erosion, Yan'gou watershed

## Abstract

At present the large scale vegetation restoration and intensive oil exploiting had brought huge influence on local environment in Yan'an region. the sediment yield data form series experiment plots and hydrological monitoring station in the Yan'an watershed after one rainfall event on July 2, 2005, which included sediment from different land uses (crop-land plot, vegetation plots, hard road surface) and 3 types roads(mountain-road brunches, mountain-road, and mountain-transport way) has been analyzed. Results showed that the erosion intensity of the 3 type roads was respectively 500 t/km^2^, 3163 t/km^2^, and 13500 t/km^2^. The sediment from cropland and grass, shrub land was within 6-184 t/km^2^. It stated that sediment from road area which only covered 1% of total area accounted for 42.3% of the total sediment yield, far beyond that from other uses of land. Sediment from grass-land and shrub-land, which covered 70.5% of watershed area, shared 26.7% of the total sediment. The further analysis showed that the 41.2% of total sediment could be detained by re-vegetation. On the contrary, that road constructing brought heavy sediment which offset the benefit of vegetation restore by 58.4%. the suggestion were to adjust our strategy from slope management to the road erosion mitigation

Many studies have confirmed it is an important measure to return the steep slope farming land to green, and to restore vegetation in line with local conditions to prevent soil erosion in the Loess Plateau [1, 2]. To implement the measure in western region, the researches on "Grain for Green", returning farmland to forest and grassland, has become popular [3, 4]. Many scholars studied the effects of farming land and trees and grass land on soil water storage [2, 5].

Tang Keli stated that the slope land for farming was the main source of the sediment in Yellow river, and the maximum gradient slope for farming land use was 25° [6]. Many authorities not only pointed out that a far-reaching influence of land-use changes on the distribution of sediment source area but also put forward some new ideas about returning farming to green in Loess Plateau [7, 8].

However, we were still not sure the contribution of returning farmland to forest and grassland on reducing sediment yield of the valley and known it was difficult to identify its contribution to the total sediment yield.

Analysis on the contribution of the stream channel and slope sediment yield had some results already [9, 10]. It was still too early to make clear the relationship between the sediment sources changes of the valley and the management.

At present, Ecological restoration in the Loess Plateau caused the sediment form the slope land declining [3]. Due to human economic activities, the mountain road developed rapidly, it is inevitable that road erosion has been intensified [11]. A. Rijsdijk and LA Bruijnzeel (1991), [12] based the valley Konto observation, pointed out that although the rural road in the area accounts for only 3% of the area, but the impact on the sediment of this area was tremendous. Nyssen J, Moneryersons J. et al (2002) [13] also think that road without protection is one of the main sources of sediment. Many kinds of protective measures have great importance to the road erosion control. So attentions were paid to the study on the protection all kind of roads.

Then what will happen to the soil erosion of the watershed, driven by the vegetation restoration and new road construction? What will happen to the proportion of sediment quality from slope land, road area and gully?

A correct understanding of the sediment sources pattern of the typical watershed is of great significance on assessment the roles of vegetation to slope management and the roles of prevention the linear path erosion.

## Introduction

The study was conducted in Yan'gou valley of the southern, Yan'an, a hilly and gully region in the Loess Plateau. Its erosion module was 6000 t/km^2^*a. The rainfall from June to September accounted for 75% of the whole year. 90% of the yearly soil erosion resulted from 3 to 5 times heavy rains or rainstorms.

The valley covers 46.88 km^2^ with a gully density of 4.8 km/km^2^, and main stream gradient of 2.41 ‰. The ecological environment has changed dramatically due to the intensive returning farmland to forest and grassland, and by the end of 2000 year, 70% of the area has been covered by forests and grasses.

The most erosion-prone slope of arable land in 1997 was 1617.6 hm^2^, and the number had been reduced to 130.7 hm^2^ in 2003. The rate of farmland reforestation had been up to 91.9%, and soil erosion intensity reduced to about 1000 t/km^2^.a [3].

Accompanying with ecological management, oil production and rural economic development, some mountain roads have been built and caused a new eco-fragile zone.

According to incomplete statistics, area of the original main mountain road (28.8 km in length, 4-6 m in width) accounts for 0.30% of the whole area, and Branch roads (1.5-2.0 km/km^2^ in length, 3 m in width) accounts for 0.50%. The new built road for oil/petroleum exploitation was 13 km in length, 6-8 m in width and occupies 0.19% of the whole areal. On the whole, there was 24% increase.

## Research methods

Comparison tests with the fixed position monitoring was applied to study on the sediment yield of the different land-use types such as oil roads, mountain roads, mountain road branches, farming land, forestry and grass area, and small managed gully, etc.

### Here's how

Selecting the road sections to be monitored: on the basis of a comprehensive investigation in the watershed, several road sections were selected which were representative roads for the oil transportation, mountain roads, and branch road (Road with vegetation), the roads were straight, flat and the surface slope of 5-12 °, the length of section is more than 80 m.

Road erosion monitoring: (1) building a pool beside the selected road to collect and measure the amount of runoff and sediment of the road. (2) measuring the gully erosion of road surface to determine the erosion of the main mountain roads, oil-transportation roads. It included Measuring the gully length, width, and depth of each gully formed by the rain, and then calculating the gully volume by the form of the triangle or trapezoidal regardless of the sheet erosion of the roads. There were 5 monitoring sections at 20-meter intervals.

Other land-uses erosion monitoring: (1) establishing runoff plots of agriculture, forest and grassland on the ubac slope at the gradient of 20-23 degrees, the plots were covered by 60% of vegetation. (2) collecting the sediment by barrels.

The sediment from steep gully area: a dam had been built to block all runoff and then to calculate the stored sediments after rainfall water by the curve of its storage capacity. The small gully side was steep, and was covered with well shrub vegetation; therefore it could on behalf of the steep slope.

Whole watershed sediment monitoring: based the valley hydrological station at the mouth of gully, by manual measuring the water level, the flow rate, samples of water and sediment at intervals in the main stream, the sediment yield of the whole watershed could be calculated out.

## Results and analysis

### The characteristics of the storm event

Over the period of July 1st and July 2nd, the precipitation ranged from 66.7 mm to 73.0 mm, which was from 13 different monitoring sites in the watershed. The rain lasted 606 min. The recorded maximum rainfall intensity by the rain gauge was 12 mm in 30 min. So, according to the criterion that the scholars developed for heavy rain in the Loess Plateau [14], the rain was a B-type rainstorm at the middle intensity whether within 30 min or in 12 hours. The recorded erosion module from the valley monitoring station was 81.3 t/km^2^ and erosion module of various types of runoff plots varied a lot.

According to the hydrological manual of Yan'an area, Yan'an meteorological station (21 years) had recorded 82.1 mm precipitation in 12 hours, the maximum rainfall intensity of 12 hours in history. the rainfall of the heaviest storm was 51.7 mm in 12 hours for annual average, the heavy rain CV value in the area was 0.51, Richard Pearson III curve (CS = 3.5CV ) shows the frequency of rain was 20%, or once a 5-year.

### Erosion intensity of different types of land-use

#### *Analysis on road erosion intensity*

##### From the main mountain roads

The measurement of main mountain road erosion caused by the rainstorm had been done on the road of 83 m in length, on which about 2 or 3 ditches appeared on the surface, and which were in near the village of the valley. The results (Table 1) showed that the average of erosion modulus was up to 16943 t/km^2^, ranging from 3163 t/km^2^ to 23348 t/km^2^. With road length downwards, due to road surface flow joining together at lower courses, a rapid increase in the erosion intensity of the road, 80 m in length on the road, sediment yield was of nearly 5 times differences. According to the fact that the road in this watershed normally used roadside catchment to collect over road flow [3, 11] the road erosion intensity at 20 meters length nearly reflect reality. The erosion of 0 ~ 20 m section represented the water-free section, was 3163 t/km^2^.

**Table 1:** Mountain road erosion analysis under the rainfall event

Length m	Slope °	Wide m	Erosion intensity (t.km^-2^)
0~20	3	6	3163
20~40	4	6	5140
40~60	5	6	13732
60~83	3	6	23348

From oil transport road: After having surveyed a road used for oil transport, with a slope of 7-8 °and 8 m wide, the results as follows: there were 3 main erosion channels; the maximum erosion was 1.92 m wide, 0.62 m deep. Excluding surface erosion, according to the method described above, the average erosion module of 0-45 m road we calculated was 13,300 t/km^2^. Lacking water catchment's diversion, and with a bigger width, the erosion of the road was serious.

From branch road: in a laced road section we set up 2 runoff and sediment measuring plots to measure the erosion intensity of non-motorized rural roads. Concrete run-off ponds were used to collect all the sediment from road surface. Results showed in Table 2: erosion intensity was 358-700 t/km^2^, an average of 530 t/km^2^. As a result, we used 500 t/km^2^ for mountain road branches.

**Table 2:** Erosion intensity analysis on two mountain road branches

Num.	Wide m	Slope °	Long m	Runoff mm	Erosion intensity t.km^-2^
1	3.0	8~9	19.8	15.4	701
2	3.5	6~7	19.9	14.7	359
mean	3.3		19.9	15.1	530

#### *Erosion intensity of different slop land types*

Sediment yield from grass land, shrub land, farmland caused by the rain was showed in Table 3. While vegetation coverage (>70% coverage) of the measuring plots were better than the average condition of the watershed. Therefore, the erosion modules of all land-uses, according to the some research results [1, 2], could be appropriately adjusted and raised. Value of sediment in forest land was approximately 10 t/km^2^, and for the grass land was 15 t/km^2^.

**Table 3:** Sediment from different landuses analysis (slope: 20-23 °)

Item	Projected size m^2^	Plot sediment g	Sediment modulus t.km^-2^
grass land	29.0	391.0	14
Grass+ shrubs	29.0	164.6	6
Farmland	25.0	4600.0	184

#### *Erosion intensity of steep slope land*

Sediment yield in the natural shrub gully (area 7.72 hm^2^ , average slope gradient > 30 °, no runoff from up-slope, vegetation coverage up to 60%~80%) lied between agricultural slope land and forest land, was 68 t/km^2^ , which was calculated from the surveyed data of the sediment yield in the dam of a gully branch. It therefore represents the erosion intensity in the steep grass land and shrub land.

#### *Erosion intensity of non-production sites*

According to the experimental data of Chabagou, Zizhou, Shaanxi, from Yellow River Water Conservancy Committee, erosion module in gully was 50%, which was higher than that form slope surface [15]. Based on land use pattern before restoration of vegetation, using area size weighted method, the sediment yield intensity of slope surface in Yan'gou watershed under this rainfall event was 77 t/km^2^. Considering that erosion intensity of gully stream bed would be higher than that of slope average, adjusted this value as 100 t/km^2^.

### The contribution of different types of land use on watershed sediment yield

Table 4 gave the rain-caused sediment intensity and area weight value of different land-use styles in the watershed.

**Table 4:** Analysis of erosion intensity under different land uses

Land use type	Sediment modulus /t.km^-2^	Area share /%	Erosion amount /t	Amount ratio /%
mountain road	3163	0.3	364	10.0
road brunche	500	0.5	120	3.3
oil road	13500	0.2	1053	29.0
grass land	15	29.1	203.8	5.6
shrub land	10	20.2	94.3	2.6
farmland	184	2.8	235.9	6.5
grass+shrub on > 35 °slope	68	21.2	672.9	18.5
un-used land	100	19.0	891.6	24.5
The total	78	93.3*	3636	100.0

* The remaining 7 percent including level terraces, dams and natural forest land.

Under the rainfall event, the total sediment yield of the watershed and the erosion intensity was 3797 t, 81 t/km^2^ respectively, which was from the monitoring Station in the gully mouth. Table 4 showed the estimated value of the total sediment yield and the intensity 3636 t and 78 t/km2, which was 4.2% less than the measured value. The calculated value well matched the measured ones, which was favor for further analysis.

Table 4 also indicated that the road area which covered only 1% of the whole watershed area yield sediment 1537 t in total, which amounted to 42.3% of total amounts of the whole watershed. So the sediment from three kind roads were 364 t from mountain road, 120 t from the mountain road branches, and 1053 t from road for oil transport, which respectively accounted for 10.0%, 3.3% and 29.0% of total sediment yield of the watershed.

Sediment yield from grass land and shrub land which covered 70.5% of the total watershed amounted to 28% of the total sediment; Grassland and shrub land above 35° slopes covered 9% of that; gully and steep slope above 35 ° with grasses and shrubs 19%; Slope for crops only 6%.

So, the road erosion could be thought as the major cause of soil and water loss in the watershed, and should be paid great attention to.

Sediment from non-production sits was 24.5% of total. In addition, about 6.7% of total from level terrace, dam land and forest which had little soil and water loss.

### The effects of "returning to green" and road construction on sediment sources

There were many findings about the soil and water reduction form restoration of vegetations. Generally, vegetation reduced more than 85% of sediment yield and 90% of water loss, comparing with the slope farm land [2, 5]. The finding in this paper also supported the above statement (Table 5).

**Table 5:** Impact of land use evolution on sediment sources in Yan'gou Watershed

Land use scenario	Slope grass+shrub %	Steep Slope grass+shrub %	Slope farmland %	Unused %	Road %	Total sediment t	Sediment modulus t·km^-2^	Reduced rate %
Year 2000	3.6	15.3	50.8	19.3	11.0	4393	94	
Year 2005	8.2	18.5	6.5	24.5	42.3	3636	78	17.3
Third pattern:	11.5	26.1	9.2	34.5	18.7	2583	55	41.2

Compared the share of sediment sources caused by three land-use patterns, Table 5 showed that in the initial management of the watershed in 2000, sediment of the watershed was mainly from slope farmlands. For the returning farmland to forest and road construction pattern: road erosion in 2005 had become a dominant factor for sediment increase of the watershed. The third pattern: assuming that after returning farm land to forest and grass, road without taking into account of newly constructed erosion, the un-utilized land and grass-shrub land would become the major source of sediment yield.

Table 5 showed the detail changes of different land contribution to sediment yield. In year 2000, sediment from farmland accounts for 50.83% of the total yield, followed by non-used land, grassland on steep slopes, and the road erosion accounted for only 11% sediment yield.

With having returned most slopes farming land to green, and without new road sediment contribution, the total sediment yield of the watershed was reduced by 41.22%, which displayed an obvious effect of soil and water conservation. The sediment sources also changed dramatically.

Sediment yield proportions in the agricultural land after a certain slope gradient returned to green was reduced to the lowest, however that from the non-production sites became the biggest, forest and grass mixed land with steep slopes also increased a lot, and the road erosion contributed larger amount as well.

With economic developing and a newly built 13-kilometer road for crude oil transport, road sediment yield had risen up to be the major role, accounting for 42.28% of the total sediment yield of the watershed. The sediment from agricultural land with a certain slope was reduced further, only accounted for 6.5% of the total. The total sediment reduction of the watershed was only 17.3% when considered the newly built road. Sediment reduction of vegetation restoration had to be compensated the road erosion increase, offset the reduction by 58.4%. The ecological effect of vegetation had been greatly counteracted.

## Conclusions and discussions

Characteristics of the soil erosion in Loess hills area are currently undergoing profound changes. Returning farmland to green and mountain roads construction have made road erosion become the dominant factor of water loss and soil erosion in the loess hills area. In an once in every 5-year frequency rainstorm event, sediment coming from road erosion accounted for 42.3% of the total sediment of the watershed, while the forest and grass mixed land which covered 70.5% of the entire watershed only contributed 26.7% of the total. Road erosion and protection methods should be paid further attention to. Although many researchers had noticed that the road erosion was one of the main sources of river silt, it was still beyond our existing recognition that the road erosion was so serious.

The different in sediment modules indicated that the various land-use types had different effects on the water loss and soil erosion in loess hilly area. The apparent distinction of the road erosion modules stated protection from road erosion should be the key contents of the ecological construction.

Watershed erosion intensity could be reduced by 41.2 percent with the help of Grain for Green, though, with the valley road and road erosion increasing. The rate of sediment reduction in the research watershed was greatly counteracted, and it was up to 58.4%, making the actual effect of the sediment reduction only 17.3%.

Through different management scenarios simulation, before the returning farmland to grass in the valley, that sediment mainly from farmland was consistent with many study conclusions, but because of the implementation of returning farmland to green, sediment from the agricultural slope land had fallen sharply. Increase of mountain road had become new pressure of environment, and it became the main sources of sediment. At the same time, due to the intense erosion of the road, the scale of the mountain road should be limited, and anti-erosion positive measures should be taken.

## Article sponsorship

The publication costs for this article were funded by Scientific & Technical Development Inc.
